# Use of Radiofrequency-Assisted Liposuction (BodyTite) for Upper Arms Lifting

**DOI:** 10.1007/s00266-023-03452-6

**Published:** 2023-06-14

**Authors:** Matilde Tettamanzi, Nicola Pili, Manuela Rodio, Pietro Luciano Serra, Claudia Trignano, Corrado Rubino, Emilio Trignano

**Affiliations:** 1 Plastic Surgery Unit, Department of Surgical, Microsurgical and Medical Sciences, University of Sassari, Sassari, Italy; 2 Department of Biomedical Sciences, University of Sassari, Sassari, Italy

**Keywords:** Upper arms lifting, BodyTite, Tumescent local anesthesia, Radiofrequency-assisted liposuction

## Abstract

**Background:**

Body contouring surgery is increasingly requested by patients, both for aesthetic and post-bariatric purposes. There has also been a rapid increase in demand for noninvasive aesthetic treatments. While brachioplasty is burdened by numerous complications and unsatisfactory scars, and conventional liposuction is unsuitable for all patients, nonsurgical arm remodeling performed with radiofrequency-assisted liposuction (RFAL) allows to effectively treat most of patients, regardless of the amount of fat and ptosis of the skin and avoiding surgical excision.

**Methods:**

A prospective study was conducted on 120 consecutive patients who presented to the author's private clinic and required upper arm remodeling surgery for aesthetic purposes or after weight loss. Patients were classified according to the modified classification of El Khatib and Teimourian. Pre- and posttreatment upper arm circumferences were taken after 6 months of follow-up to assess the degree of skin retraction obtained by treating the arm with RFAL. A satisfaction questionnaire regarding the appearance of the arms (Body-Q upper arm satisfaction) was administered to all patients before surgery and after 6 months of follow-up.

**Results:**

All patients were effectively treated with RFAL, and no cases required conversion to brachioplasty. The average reduction in arm circumference was 3.75 cm at 6 months follow-up, and patients’ satisfaction increased from 35 to 87% posttreatment.

**Conclusions:**

Radio frequency is a valid tool to treat most patients with upper limbs skin laxity, with significant aesthetic results and a high degree of patient satisfaction, regardless of the degree of skin ptosis and lipodystrophy of the arm.

**Level of Evidence IV:**

This journal requires that authors assign a level of evidence to each article. For a full description of these evidence-based medicine ratings, please refer to the Table of Contents or the online Instructions to Authors www.springer.com/00266.

## Introduction

Removal of excess fat in various areas of the body has always been a desire and a goal in western countries, where obesity rates are constantly increasing in the population. In literature, many procedures for body remodeling surgery are described, both for aesthetic purposes and after major weight loss. These procedures are shown to improve body image and, therefore, patients’ quality of life [1, 2]. The increase in subcutaneous fat and progressive tissue laxity make the upper arm remodeling surgery one of the most requested and challenging simultaneously. Two corrective options are available to face upper arm remodeling: excisional dermolipectomy and nonsurgical techniques such as radiofrequency-assisted liposuction (RFAL). Brachioplasty has always represented the gold standard for patients with skin laxity and consists of surgical removal of excess fat and skin. Although this is a very effective surgical procedure, brachioplasty is burdened by complications [3] such as hematomas, seromas, infections, and the poor acceptance of the extensive and visible scars that can affect patient satisfaction [4]. For this reason, nonsurgical techniques have become increasingly popular in upper limb remodeling due to a rapid increase in the demand for noninvasive aesthetic treatments. Since 2012, the number of performed noninvasive procedures has grown by more than 200% [5]. Conventional liposuction, although one of the most performed procedures in cosmetic surgery [6], is unsuitable for all patients. It is particularly indicated in patients with significant excess fat but minimal skin laxity. Ideally, the best technique for patients with arms skin laxity would be the one allowing to tighten the skin and remove excess fat at the same time, avoiding long scars and with minimal complications. This work describes our case history of nonsurgical arm remodeling performed with radiofrequency-assisted liposuction (RFAL). Our study aims to demonstrate how most cases of upper arm remodeling can be effectively treated by avoiding surgical brachioplasty and all its consequences.

## Methods

From 2016 to 2021, 120 patients underwent arm remodeling with radiofrequency-assisted liposuction (RFAL). All procedures were performed in the author’s accredited outpatient clinic. The surgical team comprised a board-certified plastic surgeon, an assistant surgeon, an operating room nurse, and a board-certified anesthesiologist. All patients selected for the study were informed about the procedure and signed consent was obtained. Patients who needed improved upper arm contours were categorized according to El Khatib [7] and Teimourian and Malekzadeh system [8]. A single adaptation has been made to the classification: the vertical height ptosis measurement was changed with the arm circumference measurement at the point of maximum ptosis. One week before the surgery and after 6 months of follow-up, the "BODY—QTM—satisfaction with upper arms" questionnaire was administered to each patient and the degree of satisfaction with the appearance of the arms was assessed. The posttreatment result was compared with that before surgery to evaluate any improvement following treatment. A pretreatment screening was performed in each patient, measuring the upper arm circumference at the point of maximum skin ptosis. This measurement was detected with the arm raised in line with the shoulder, and the forearm flexed to 90° forming a 90° angle between the arm and the chest.

Patients were treated in the upper arms using the BodyTite RFAL device. The BodyTite’s handpiece has two electrodes: an internal cannula emits simultaneous energy and suction in radio frequency (RF) and an external electrode reflects heat in the dermis. The RF energy causes a contraction of the fibrous septae surrounding the fat globules. When delivered at stratified depths, this RF energy tightens the connection of the skin and fat layer to the underlying fascia and the overlying dermis. This methodology allows the improvement of skin laxity in the upper arms region.

BodyTite also delivers double-sided skin heating. The handpiece features a unique external thermistor directly above the internal RF cannula tip; so directional heating occurs only between the cannula tip and the external electrode, which minimizes seroma formation [9].

The cutaneous surgical incision sites are infiltrated with 1% lidocaine with 1:100,000 epinephrine. The cannula is inserted through three incisions: the first one is made 1 cm above the humeral epicondyle, the second one is made 2 cm anterior to the posterior axillary fold, and a third incision is made between the previous two. Every patient underwent tumescent local anesthesia: the tumescent solution was prepared with 25 mL of 2% lidocaine, 8 mEq of sodium bicarbonate, and 1 mL of epinephrine (1 mg/1 mL) in 1000 mL of 0.9% saline solution [10–12]. Overall, 180–250 mL were introduced per arm. After the TLA infusion, the first incision was made 20–40 min later to allow epinephrine and lidocaine to have their effect. We used a thin 3 mm cannula and adopted a “crisscross” pattern of aspiration. This pattern allows the reduction of the degree of postoperative contour irregularities and the appearance of linear cannula lines by breaking the fibrous septae at the level of the subcutaneous fat. In this phase aspiration was not performed. Subsequently, liposuction is carried out with the "Liposurg" using a 3 mm cannula at the level of the deep layer of the aspirated subcutaneous fat leaving a fatty layer of at least 5 mm from the dermis. After aspirating the subcutaneous fat, the use of a basket cannula allows centrifuging the fat and levels the areas previously treated, redistributing the remaining fat and avoiding contour irregularities, depressions and undulations of the skin, which are the most frequent complications of liposuction [13]. Eventually, we use the BodyTite cannula. The area to be treated must be covered with gel. We set 38 °C as cutoff for skin temperature, 9–12 kJ per arm, and 20 W. The achievement of the temperature and kilojoules cutoff for each individual area gives the end point of the treated area with the radio frequency. After the treatment, the cutaneous incisions are sutured with 5/0 Nylon thread and compressive dressings are applied for 30 days. On average, 80–120 cc of fat per single upper limb is aspirated with the internal cannula. The goal of deeper heating was to obtain contraction of fibrous septae and generate punctuate adhesions of the fat/skin complex to the underlying fascia [9]. Follow-up was fixed at 1 month and 6 months after surgery. Skin laxity was measured 6 months after surgery and compared with before treatment. Patients who refused to be followed up, pregnant or breastfeeding women, and patients with previous liposuction procedures, surgery, or lipolysis injection in the upper arm region were excluded from the study.

## Results

During 5 years, we analyzed 120 patients, 96 women and 24 men, who underwent radiofrequency-assisted liposuction. All procedures were performed using the TLA technique.

The median (IQR) age at surgery was 44 years (29–62), and the mean BMI was 28 (range 25–31). The average amount of tumescent solution infiltrated was 200 mL (180–250 mL). No signs of adrenaline or lidocaine toxicity were reported.

According to El Khatib classification, most patients undergoing radiofrequency-assisted liposuction (RFAL) were classified as stage 2B (42%). Four patients were classified in stage 1 (3.5%), thirty-five patients in stage 2A (29%), fifteen in stage 3 (12.5%), and sixteen in stage 4 (13%) (Table 1).

**Table 1:** Patients were classified according to El Khatib classification

Stage (El Khatib classification)	No. of patients	Percentage
1	4	3.5
2A	35	29
2B	50	42
3	15	12.5
4	16	13

Our case studies found that the average circumference of the upper limb at the point of maximum skin ptosis was 27.3 cm before treatment. An average of 100 mL of fat were removed for each upper arm. After treatment, at 6 months follow-up, we detected 23.55 cm as the average measurement of the upper limb circumference. We noticed how the circumference of the limb was reduced by 75% after the first month, then reached the maximum reduction after 6 months and eventually stabilized. A comparison between preoperative and postoperative satisfaction was obtained by “Body-Q satisfaction with upper arm” questionnaire. We reported an average satisfaction rate of 35% before treatment, a value of 87% at the sixth month of follow-up and a percentage increase between the two values of 149% (Table 2).

**Table 2:** “Body-Q satisfaction with upper arm” questionnaire results

No. of patients	% Satisfaction (pretreatment)	% Satisfaction (posttreatment)	% Increase
120	35	87	149

One of the most interesting data from our study is the number of patients who required conversion to surgical excision of skin excess after RF treatment. In our series, no patient required brachioplasty, and in 10 cases (8.3%) it was necessary to improve the result by repeating the treatment with RF after about 1 year. Overall, our trend goes against the rate reported in the literature, where the need for brachioplasty is recommended starting from El Khatib classification stage 2B [7].

Among the major complications associated with liposuction, it is possible to find in literature hyperesthesia, pain, skin hyperpigmentation, hematoma formation, seroma formation, infections, chronic swelling, and skin slough [13]. Unattractive access scars, a lumpy or irregular skin surface, and residual skin laxity can also occur. Furthermore, the passage of the cannula in a plane that is too superficial and, therefore very close to the skin can cause dermal burn or depression [9]. We reported 10 cases (8.3%) of hyperesthesia which resolved in the first 6 months. No hematoma, skin slough, chronic swelling, or pain cases were reported. Two patients (1.6%) presented seroma formation. There have been five cases of fat necrosis (4.2%). Sixteen patients (13.3%) noted residual skin laxity which required a revision 1 year after surgery, but in no case was brachioplasty performed (Table 3).

**Table 3:** Complications

Complications	No. of patients	Percentage
Hyperesthesia	10	8.3
Hematoma	0	0
Irregular skin surface	0	0
Chronic swelling	0	0
Pain	0	0
Seroma	2	1.6
Scar revision	0	0
Fat necrosis	5	4.2
Residual skin laxity	16	13.3

## Discussion

In this article, we present our experience of 120 consecutive cases of lifting of the brachial region with radiofrequency-assisted liposuction (RFAL) over 5 years time. Our complication rate was 27.5%, including hyperesthesia (8.3%) and fat necrosis (4.2%). In sixteen cases, a revision for residual skin laxity was necessary. No major complications were recorded, and conversion to surgical brachioplasty was not required.

Lifting of the brachial region is a challenging procedure and presents numerous complexities. On one side, the patients' expectations are high, looking for important changes in terms of size and skin characteristics. On the other side, the surgeon must deal with skin which has often altered characteristics in terms of laxity and striae. Furthermore, the real challenge is skin laxity, as the size reduction of the arm is not enough to satisfy the patient.

Patients are induced to undergo this type of procedure to expose their arms without problems, without the annoying pendulous skin moving with every movement of the limb. For this reason, reducing the arm size through major liposuction without remodeling the subcutaneous tissue and the skin would not allow for a satisfactory aesthetic result.

Pitman and Teimourian [14] noted that 21.7% of patients undergoing arm liposuction had unsatisfactory results, and in the majority of cases, this is attributable to excess skin, which remains after the treatment. The authors argue for a surgical resolution with an excision of the excess cutaneous tissue. The arm, however, is a very particular area where brachioplasty scars are particularly visible and impact the final result of the procedure.

In a more recent study, Di Pietro et al. [15] accounted poor satisfaction in one out of four patients undergoing brachioplasty due to extensive and visible scars.

Gasperoni et al. [16] claimed a substantial skin retraction by performing full-thickness liposuction of the subcutaneous fat, including both the deep and the superficial layers. Liposuction causes a reduced volume of the limb accompanied by a cicatricial retraction of the skin, but this retraction is not sufficient compared to the one obtained with radio frequency. A simple circumferential aspiration is only based on the reduction of the fat volume and the retraction caused by the scar. In our opinion BodyTite, having a constant and controlled thermal effect on the treated area, determines a more significant skin retraction which is not obtainable with liposuction alone. The objective of our study was to demonstrate that every grade of ptosis of the arm can be corrected by using BodyTite, whatever the condition of skin laxity. No patient in our case history required surgical brachioplasty following radiofrequency treatment. Our BodyTite technique allowed us to have a 13.7% reduction in the circumference of the arms. The possibility of concentrating the RF between the two electrodes, a smaller internal one at the level of the subcutaneous fat and a larger external one in contact with the skin, allowed a selective treatment of a particular body area. Specifically, the temperatures reached are higher in the inner layer and lower on the skin where the outer electrode is present. This technique allows, with a high internal temperature, to coagulate the subcutaneous fat and causes a scar reaction at the level of the fibroseptal network resulting in skin contraction and avoiding skin burns thanks to the low external temperature. It also allows for high efficiency and control of the energy that is only radiated between the two electrodes and avoids the need for grounding. The device measures the amount of energy used in the single treatment area and the temperature on the skin surface in real time. Once the temperature set in the machine is reached, the acoustic alarm allows to stop the treatment on one skin area and move to the adjacent one.

In several studies, the endpoint has been evaluated based on the characteristics of the treated area in terms of lack of resistance, palpable warmth, and mild erythema [9]. In our opinion, this can lead to the uneven treatment of the different areas. We believe BodyTite, being an operator-dependent method and using the parameters of temperature and energy released on the single treated area, reduces the probability of error and permits a uniform treatment regardless of the operator performing the treatment.

Another critical issue we found in the literature is the definition of the reduction of skin contraction. Some authors evaluate the distance between two well-defined skin marks, such as pigmented lesions, scars, or landmarks, to evaluate skin contraction. The pinch test is also widely used, in which the operator grasps the ptotic skin hanging below the bicipital sulcus to assess skin ptosis. However, the pinch test is an imprecise and operator-dependent parameter. Moreover, skin fold calipers can measure the thickness of the skin and subcutaneous fat by approximating the two prongs.

This method carries variability: as a matter of facts, a firm pinch creates a “thinner” measurement, while a looser approximation will create an apparently thicker subcutaneous measurement.

In an interesting study by Irvine [9], to assess postoperative skin contraction, each patient was preoperatively marked with a tattoo on the internal surface of the arm. At 1-year follow-up, they calculated the degree of skin surface area contraction based on the tattoo markers by comparing preoperative and postoperative values for four treatment areas: the proximal and distal right volar upper arms and proximal and distal left upper arms. In our opinion, this method is preferably not applicable, as we want to obtain from BodyTite treatment a 360° reduction of the arm circumference and not only a four areas treatment. Furthermore, the tattoo represents an indelible mark which persists on the skin, and we prefer not to propose it to a patient undergoing an aesthetic treatment. Our scars are poorly visible, and, in most cases, only two accesses are needed at the level of the humeral epicondyle and the armpit, a third access is required only in cases of particularly long arms.

In literature, laser-assisted liposuction (LAL) is another widely described methodology. LAL uses a device with a helium–neon energy source used for lipoplasty. The energy source comes from the tip of a bare laser fiber directed at the subcutaneous level to lase and suction fat [17].

The laser beam forms a sort of cone at the point where it is applied, so the energy is concentrated on the tip of the system, which can have a working radius of 90°–180° depending on the fiber used.

Both Dudelzak [18] and Prado [19] compared their studies' efficacy of laser-assisted lipoplasty versus suction-assisted lipoplasty. In particular, Prado et al. subjected the patients to laser-assisted lipoplasty on one half of the body, and in the contralateral side they applied suction-assisted lipoplasty in more comparable areas of the body. In both studies, no major clinical differences or discrepancies in terms of results for suction-assisted lipoplasty versus laser-assisted lipoplasty were found.

Similarly, Pereira-Netto et al. [20] reached the same result in a more recent study, claiming that it is not possible to support with a high level of reliability that laser-assisted lipoplasty is superior to traditional liposuction. This could be explained because being the radio frequency a bipolar, the energy necessarily passes between the two electrodes, an internal positive pole and an external negative one. This means no energy is transmitted from the internal cannula toward the inner layer of the tissues, making the treatment easily manageable and reducing tissue damage. The energy necessarily needs to pass outwards between the internal and external electrodes on the skin, allowing to treat the tissue at full thickness and consequently having better results in terms of skin retraction.

Another upper extremity treatment technology is the VASER liposuction (Sound Surgical Technologies, LLC, Louisville, Col.) [21], which uses an ultrasound system specifically directed on adipocytes. The ultrasound beam causes an emulsification of the fat which is eventually aspirated. In particular, the target of this method is the water contained in the adipocytes. Given that this method has not an action at the level of the fibro septal network, skin retraction does not occur. Consequently, the various degrees of retraction described are only attributable to lipolysis. For this reason, VASER technology is consolidated when adding a second method which allows for the retraction effect.

A recently introduced device is the Renuvion/J-Plasma helium-based plasma device [22], which associates radio frequency and an inert gas such as helium to cause a very selective contraction of soft tissues. This device makes it possible to heat the fabric up to 85 °C within 0.05 s. Unlike every other method, the tissue surrounding the treated area has much lower temperatures, allowing a selective treatment with a lower risk of injury to the surrounding tissues. Firstly introduced in 2018, the Renuvion/J-Plasma technology seems to achieve earlier results than the BodyTite, but the outcomes are comparable in the long term.

In a recent study, Katz [23] demonstrated how procedures delivering high intensity focused electromagnetic field energies and RF simultaneously on multiple body areas could be an effective and comfortable treatment for fat reduction on multiple body parts, thickening of underlying muscles, and overall improved aesthetic appearance. The study targeted the abdomen, saddlebags, inner thighs, and buttocks. Still, our goal was to reduce and tighten the subcutaneous fat layer of the upper extremities with RF, without the need to thicken the muscles with the addition of HIFEM.

Most of our surgeries have been performed for cosmetic reasons. In many cases, patients seek body reshaping to remove excess skin after major weight loss. Whether performed for cosmetic purposes or after weight loss, body contouring can potentially improve the patient's body image and health-related quality of life (HRQL) [24, 25]. We administered the BODY-Q questionnaire to our patients to assess the impact on quality of life and personal image of the appearance of the arms.

The BODY-Q tool consists of different modules to measure patients reported outcomes and satisfaction following surgery. There are body contouring-specific modules (abdomen, chest, buttock), and the upper arms satisfaction scale was administered to our patients. This scale includes seven items which must be evaluated by the patient using a score from 1 to 4 (1 very dissatisfied, 4 very satisfied) regarding shape, size, skin characteristics, aspect of the limb when dressed with short-sleeved clothes, etc. The response rate was 100% both pretreatment and at 6 months follow-up.

**Fig. 1:**
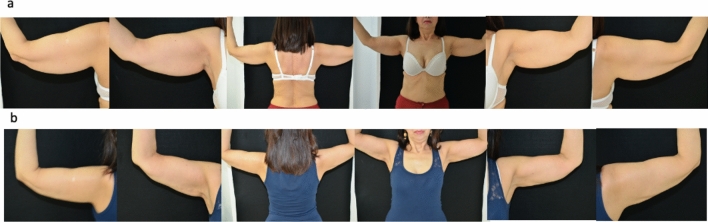
**a** Preoperative view. **b** Postoperative view after 6 months

**Fig. 2:**
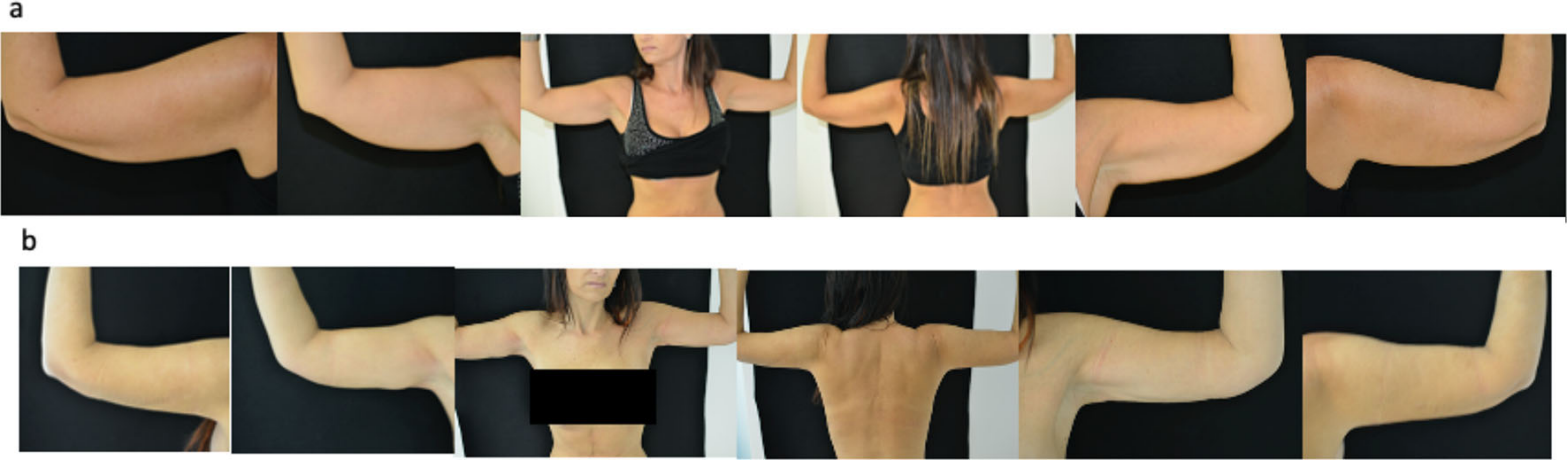
**a** Preoperative view. **b** Postoperative view after 6 months

From our results, it is evident how the appearance of the upper extremities, particularly in women, has a great impact on body image and psychophysical well-being. Large and flabby arms are perceived as unsightly and give an unpleasant impression to an individual physical appearance. On the other hand, slender arms recall a feminine and refined image. Furthermore, the upper extremities are heavily involved in relationship life and in nonverbal language. For all these reasons, the improvements obtained with RF were perceived by our patients as strongly positive, with an increase in satisfaction after treatment of 149% (Figs. 1, 2). Eventually, patient reported outcomes (PRO) are also needed to ensure that cosmetic body contouring procedures, including nonsurgical treatments, are safe and effective. Many times physicians consider satisfactory a result which may not be enough for the patient, but usually expectations regarding body contouring surgery are very high and might be difficult to achieve.

## Conclusion

According to the American Plastic Surgery Society [26] 18,000 arm lifting procedures were performed in 2017. Although the number is steadily increasing, brachioplasty is fraught with numerous complications. In addition to the risk of infections, seroma formation and wound dehiscence, the most important complication that limits its widespread use is residual scars. This procedure involves a straight scar from the armpit to the elbow which is difficult to hide. In the arm lifting procedure, a less optimal result in terms of residual ptosis but with less evident scars is preferable to a perfect lifting with showy scars. Radio frequency allows to treat the majority of patients who require an arm lifting through a safe, reproducible method and with high levels of patient satisfaction.

## References

[1] Pavan C, Marini M, De Antoni E, Scarpa C, Brambullo T, Bassetto F, Mazzotta A, Vindigni V (2017). Psychological and psychiatric traits in post-bariatric patients asking for body-contouring surgery. Aesthet Plast Surg.

[2] Monpellier VM, Antoniou EE, Mulkens S, Janssen IMC, van der Molen ABM, Jansen ATM (2018). Body image dissatisfaction and depression in postbariatric patients is associated with less weight loss and a desire for body contouring surgery. Surg Obes Relat Dis.

[3] Nguyen L, Gupta V, Afshari A, Shack RB, Grotting JC, Higdon KK (2016). Incidence and risk factors of major complications in brachioplasty: analysis of 2,294 patients. Aesthet Surg J.

[4] Ngaage M, Agius M (2018). The psychology of scars: a mini-review. Psychiatr Danub.

[5] American Society of Plastic Surgeons (2017). 2017 plastic surgery statistics report. Plast Surg.

[6] Pallua N, Wolter T (2011) Liposuktion [Liposuction]. Chirurg 82(9):759–760, 762–764, 766. 10.1007/s00104-011-2106-8. **(in German)** 10.1007/s00104-011-2106-8 21826569

[7] El Khatib HA (2007). Classification of brachial ptosis: strategy for treatment. Plast Reconstr Surg.

[8] Teimourian B, Malekzadeh S (1998). Rejuvenation of the upper arm. Plast Reconstr Surg.

[9] Duncan DI (2012). Improving outcomes in upper arm liposuction: adding radiofrequency-assisted liposuction to induce skin contraction. Aesthet Surg J.

[10] Bolletta A, Dessy LA, Fiorot L, Tronci A, Rusciani A, Ciudad P, Trignano E (2019). Sub-muscular breast augmentation using tumescent local anesthesia. Aesthet Plast Surg.

[11] Trignano E, Tettamanzi M, Liperi C (2023). Outcomes of intramuscular gluteal augmentation with implants using tumescent local anesthesia. Aesthet Plast Surg.

[12] Trignano E, Serra PL, Pili N, Trignano C, Rubino C (2022). Hybrid breast augmentation: our surgical approach and formula for preoperative assessment of fat graft volume. Gland Surg.

[13] Bellini E, Grieco MP, Raposio E (2017). A journey through liposuction and liposculture: review. Ann Med Surg (Lond).

[14] Teimourian B, Rogers WB (1989). A national survey of complications associated with suction lipectomy: a comparative study. Plast Reconstr Surg.

[15] Di Pietro V, Colicchia GM, Cervelli V, Gentile P (2018). Arm Contouring after massive weight loss: liposuction-assisted brachioplasty versus standard technique. J Cutan Aesthet Surg.

[16] Gasperoni C, Salgarello M (1994). MALL liposuction: the natural evolution of subdermal superficial liposuction. Aesthet Plast Surg.

[17] Zelickson BD, Dressel TD (2009). Discussion of laser-assisted liposuction. Lasers Surg Med.

[18] Dudelzak J, Hussain M, Goldberg DJ (2009). Laser lipolysis of the arm, with and without suction aspiration: clinical and histologic changes. J Cosmet Laser Ther.

[19] Prado A, Andrades P, Danilla S, Leniz P, Castillo P, Gaete F (2006). A prospective, randomized, double-blind, controlled clinical trial comparing laser-assisted lipoplasty with suction-assisted lipoplasty. Plast Reconstr Surg.

[20] Pereira-Netto D, Montano-Pedroso JC, Aidar ALES, Marson WL, Ferreira LM (2018). Laser-assisted liposuction (LAL) versus traditional liposuction: systematic review. Aesthet Plast Surg.

[21] Nagy MW, Vanek PF (2012). A multicenter, prospective, randomized, single-blind, controlled clinical trial comparing VASER-assisted lipoplasty and suction-assisted lipoplasty. Plast Reconstr Surg.

[22] Gentile RD (2019). Renuvion/J-plasma for subdermal skin tightening facial contouring and skin rejuvenation of the face and neck. Facial Plast Surg Clin N Am.

[23] Katz B (2023). Concomitant use of radiofrequency and high intensity focused electromagnetic field energies for full-body remodeling: MRI evidence-based prefatory trial. J Cosmet Dermatol.

[24] De Zwaan M, Georgiadou E, Stroh CE, Teufel M, Köhler H, Tengler M, Müller A (2014). Body image and quality of life in patients with and without body contouring surgery following bariatric surgery: a comparison of pre- and post-surgery groups. Front Psychol.

[25] Song AY, Rubin JP, Thomas V, Dudas JR, Marra KG, Fernstrom MH (2006). Body image and quality of life in post massive weight loss body contouring patients. Obesity (Silver Spring).

[26] Cosmetic Surgery National Data Bank Statistics (2018) Aesthet Surg J 38(suppl_3):1–24. 10.1093/asj/sjy132 10.1093/asj/sjy132 29846503

